# A Novel Sensible Smart Mask Using Micro Thermal-Electric Energy Conversion Elements †

**DOI:** 10.3390/mi15080991

**Published:** 2024-07-31

**Authors:** Yun Zhang, Zonglin Xiao, Binggang Liu, Xiaoming Ren, Cirui Liu

**Affiliations:** 1 State Key Laboratory of Electromechanical Integrated Manufacturing of High-Performance Electronic Equipments, Xidian University, Xi’an 710071, China; 2 School of Mechano-Electronic Engineering, Xidian University, Xi’an 710071, China; 3 AVIC Xi’an Flight Automatic Control Research Institute, Xi’an 710065, China; 4 Science and Technology on Applied Physical Chemistry Laboratory, Shaanxi Applied Physics and Chemistry Research Institute, Xi’an 710061, China

**Keywords:** sensible smart mask, thermal-electric energy conversion, thermoelectric generator module, respiratory rate sensing

## Abstract

In poor sanitary conditions, people need to wear masks to protect the health of their respiratory system. Meanwhile, it is necessary for patients with respiratory diseases to have real-time measurement on respiratory rate when wearing masks. Thermoelectric generation provides a new approach and method for powering and sensing small low-power devices, and has good application prospects in smart masks. In view of this, a novel sensible smart mask using micro thermal-electric energy conversion elements (TECE) is proposed in this paper, which can detect and display the respiratory rate in real time. First, the temperature conversion characteristic of micro TECE represented by the thermoelectric generator module is analyzed. Second, the respiratory characteristics of the human body are studied, and the respiratory rate sensing effect based on micro TECEs is analyzed and verified. Then, a sensible smart mask, which can show respiratory rate in real time, is developed by integrating MCU and OLED module. Finally, human respiratory rate experiments are conducted, the experimental results verified the effectiveness and accuracy of the proposed sensible smart mask.

## 1. Introduction

Respiratory rate is one of the important vital signs that is able to respond to the health condition of human body [1]. The COVID-19 pandemic is a growing public health concern worldwide. It causes infection in the lower respiratory tract, which can lead to alveolar damage and respiratory failure in severe cases [2]. Respiratory rate is a common indicator for judging lower respiratory tract infections in the clinic, and extensive research has shown that the risk of COVID-19 pandemic infection can be predicted by analyzing the change of respiratory rate [3]. Real-time detection of respiratory rate is a guarantee of life safety for various respiratory disorders such as asthma, and a portable respiratory rate detection device can also be of great use in home detection for patients with respiratory diseases. Therefore, it is crucial to develop a wearable device that can measure respiratory rate in real time. However, wearable respiratory rate detection devices applied at this stage, are usually realized by detecting the fluctuation frequency of the human thorax, which requires the use of sensors fixed on wristbands or clothes [4,5], resulting in low detection accuracy, high costs, and limited scalability for widespread adoption.

Wearing masks is the simplest and most efficient strategy to restrict the progression of the COVID-19 pandemic and other infectious diseases [6]. Droplet and contact transmission are the two main routes of transmission for the COVID-19 pandemic. Infected people’s droplets will produce aerosols in the air, while masks can minimize the amounts of droplets emitted by wearers, effectively inhibiting virus transmission [7]. Through mathematical modeling, Li et al. indicate that wearing a face mask can be effectively combined with social distancing to flatten the epidemic curve, wearing a mask presents a rational way to combat COVID-19, and more countries and regions are moving forward with recommendations or mandates to wear masks in public [8]. Therefore, mask-based wearable respiratory monitoring devices take into account the needs of epidemic prevention and health monitoring.

The Information contained In one’s breath is also a significant aspect in determining one’s health, and the mask is close to the face and serves as an excellent carrier for respiratory sensors. As a result, a mask that continuously monitors the user’s respiratory state in daily life is required for tailored medical care and epidemic prevention. Zhong et al. have integrated the self-powered pressure sensor with the mask to produce an intelligent mask [9]. Under the influence of respiratory gas, the pressure sensor can generate a peak voltage of roughly 10 V. Nguyen et al. attached a flexible airbag to a mask and used a pressure sensor to measure the flexible airbag’s pressure change in order to measure the pulse, blink, and respiration signals [10]. He et al. used the friction nano generator as the sensor of the smart mask to detect various respiratory indicators such as respiratory rate and inspiratory time [11].

In addition to the pressure characteristics of breathing, the researchers also measured the respiratory rate through the temperature characteristics of breathing. Xue et al. used pyroelectric materials to make a self-powered sensor installed on the mask, which can convert the temperature change in breathing into electrical energy and reflect the respiratory rate of human body [12]. Thermoelectric generators have also been extensively researched for detecting respiratory characteristics. Compared to other materials, a thermoelectric generator has the advantages of high conversion efficiency, high output power and high service life. Goto et al. proposed a micro-thermoelectric gas sensor for selective gas detection in breath, the thermoelectric voltage of the sensor is induced by the catalytic combustion of hydrogen or methane. Their work was conducted under an elevated temperature, which used a micro-heater built on the same membrane as a hotplate, and then enabled selective combustion of the target gas [13]. Based on the literature review, it can be concluded that the effective measurement of respiratory rate can be achieved by utilizing the body’s own energy sources.

Although the above-referenced studies achieved notable results, the mentioned mask with embedded sensors may require high temperatures or have a large sensing area, which is not conducive to flexible use. Aiming to solve the problem, we have developed a novel sensible smart mask using micro thermal-electric energy conversion elements (TECE). The primary work of this paper are as follows: the relationship between TECE and temperature difference is verified, a smart mask integrating TECE, MCU, OLED screen and other modules has been proposed, achieving automatic measurement of respiratory rate at room temperature, and offering advantages in compact size, lightweight, and facial conformity.

The paper is structured into five sections. Following the introduction, the conversion characteristic of micro TECE is analyzed in Section 2, which indicates the linear relationship between the output voltage of TECE and the temperature difference. Section 3 analyzes the fundamental characteristics of breath and gives the output law of TECE due to human respiration. In Section 4, describes the development of a sensible smart mask by integrating MCU and OLED modules, and this mask can show respiratory rate in real time. The effectiveness of the proposed mask is verified by experiments. Finally, the conclusions are given In Section 5.

## 2. Conversion Characteristic of Micro TECE

The conversion principle of micro TECEs, represented by thermoelectric generator, is the Seebeck effect. This effect arises when a temperature difference is applied between two coupled conductors or semiconductors. It forms the theoretical foundation for TECEs as respiratory sensors. When different temperatures are applied to the two ends of TECE, the Seebeck effect results in a thermoelectric voltage [14]. Therefore, by measuring the temperature difference inside and outside the mask caused by breathing, respiratory rate can be achieved by processing the voltage signal of the TECE.

The Peltier effect also influences the output voltage of the TECE. Depending on the direction of the current flow, heat absorption and release occur at the junctions of different conductors [15]. Therefore, when TECE is utilized as a temperature sensor, this effect leads to the loss of electrical energy converted from thermal energy, and then reducing the output voltage.

(1) $$V=(S-U)\cdot (T_{h}-T_{c})\cdot N$$

where *S* is the Seebeck coefficient of TECE arm material, *U* is the Peltier coefficient, *T_h_* is the hot end temperature, *T_c_* is the cold end temperature, and *N* is the number of thermoelectric arms.

**Figure 1:**
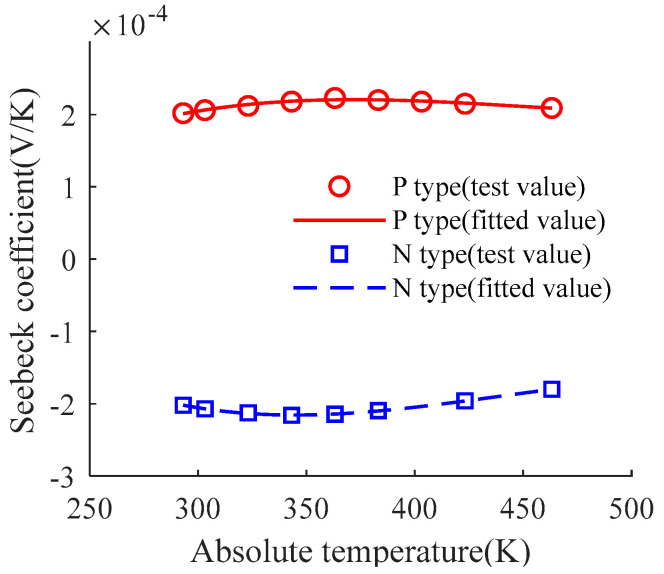
Seebeck coefficient of thermoelectric arm material.

Figure 1 shows the curves of Seebeck coefficient of P-type and N-type material produced by TECE changing with temperature. The Seebeck coefficient represents the thermoelectric conversion efficiency of the TECE, which is almost unaffected by temperature changes. When the logarithm of the material and thermoelectric arm is constant, the change of the output voltage is only related to the temperature difference between two sides of the TECE, implying that output voltage and temperature difference have a one-to-one mapping relationship.

In order to obtain effective thermoelectric properties, the Seebeck coefficient and Peltier coefficient are usually processed by integrating and normalizing over the temperature gradient. Then, the normalized Seebeck coefficient and Peltier coefficient can be regarded as a constant, which means that TECE has good linearity when used as a temperature sensor.

(2) $$S_{p,eff}=\frac{\int_{T_{c}}^{T_{h}}S_{p}\left(T\right)dT}{T_{h}-T_{c}}$$

(3) $$S_{n,eff}=\frac{\int_{T_{c}}^{T_{h}}S_{n}\left(T\right)dT}{T_{h}-T_{c}}$$

(4) $$U_{n,eff}=\frac{\int_{T_{c}}^{T_{h}}U_{n}\left(T\right)dT}{T_{h}-T_{c}}$$

(5) $$U_{p,eff}=\frac{\int_{T_{c}}^{T_{h}}U_{p}\left(T\right)dT}{T_{h}-T_{c}}$$

(6) $$S=S_{p,eff}-S_{n,eff}$$

(7) $$U=U_{p,eff}-U_{n,eff}$$

**Figure 2:**
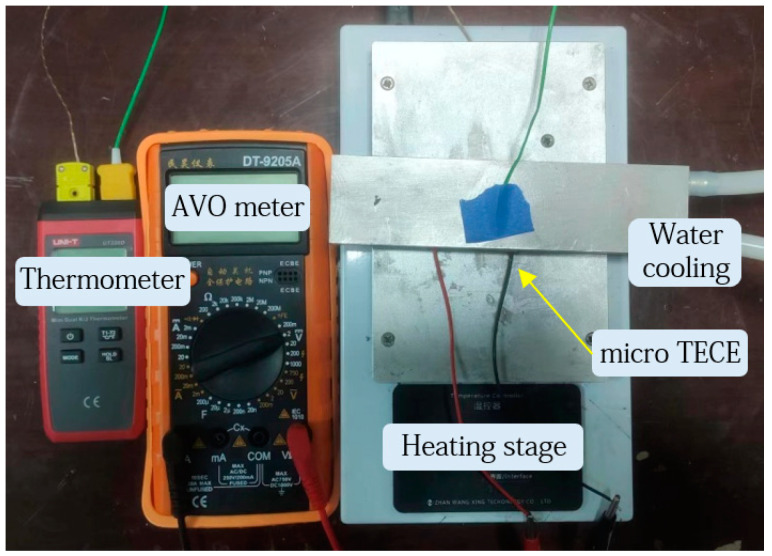
TECE performance testing platform.

To investigate the steady-state output performance of TECE, various temperature differentials are applied to the TECE, its output voltage are measured in detail and compared with the calculated outcomes. To effectively conduct experiments, as shown in Figure 2, a TECE performance testing platform is designed and built, which mainly includes the TECE, a constant temperature heater serving as the heat source, a water-cooling system providing the cold source, an AVO meter, and a thermometer.

The TECE utilized in the experiment has a length and width of 15 mm and a thickness of 3.6 mm. The size of a single thermoelectric arm is 1 mm × 1 mm × 2 mm, and each TECE is combined 31 thermoelectric arms. Based on the experimental platform, the output performance of TECE was tested and compared with the simulated results.

**Figure 3:**
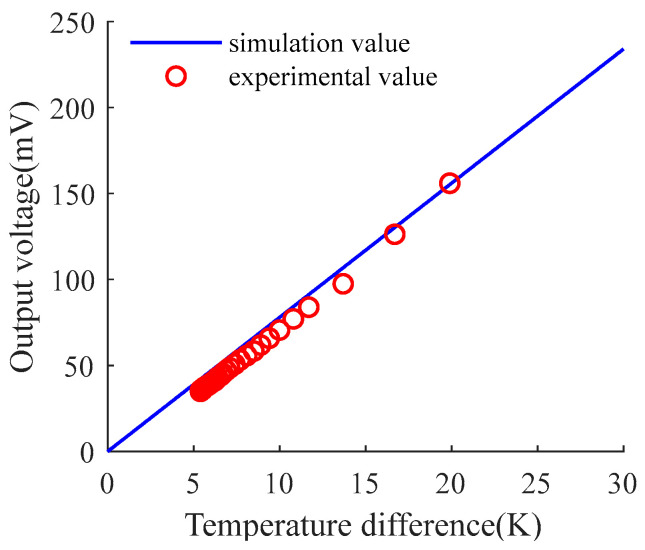
Comparison between experimental and calculated values of thermoelectric generator.

As shown in Figure 3, the experimental and simulated values of the TECE are in good agreement. The temperature difference and the output voltage have a linear relationship. If the temperature on one side of the TECE is known, the temperature difference between the two sides can be calculated according to the output voltage, so as to realize the linear measurement process of the temperature on the other side.

## 3. Sensing Effect of Respiratory Rate

**Figure 4:**
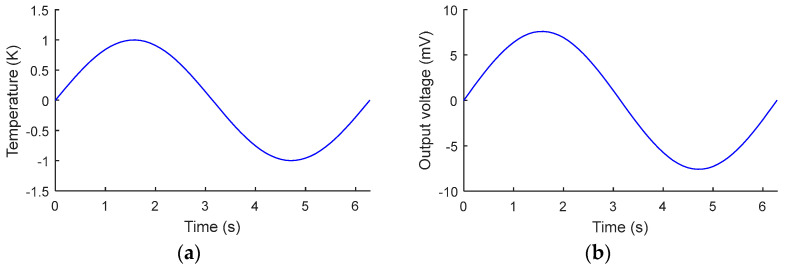
Calculation of periodic temperature difference signal and its output voltage. (**a**) Sinusoidal temperature difference signal. (**b**) Output voltage generated by sinusoidal signal.

When a TECE is used to measure respiratory rate, it can be regarded as applying a periodic temperature difference change to TECE. In the simulation, a time sinusoidal varying temperature difference is imposed for the TECE, as shown in Figure 4a. As shown in Figure 4b, the TECE exhibits robust responsiveness to periodic variations in temperature difference, correlating directly with changes in output voltage. This characteristic makes it suitable for application as a breathing temperature sensor.

**Figure 5:**
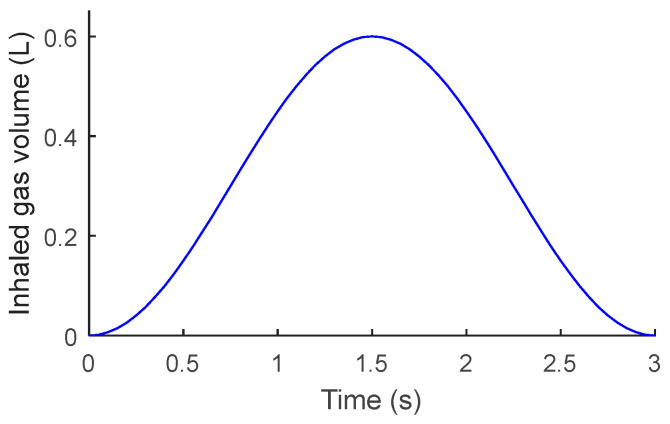
Inhaled gas volume.

The tidal volume of normal adults is 8–10 mL/kg and the respiratory rate is 16–20 times/min [16]. For an adult, if the weight is 60 kg, the tidal volume of one breath is 0.6 L and the respiratory cycle is 3 s. The change in volume of the inhaled gas can be approximated as a cosine function, as shown in Figure 5. There exists a relationship between the volume of inhaled gas and time as follows:

(8) $$V(t)=0.3\cos(\frac{2\pi }{3}t+\pi )$$

If the area of nasal outlet is *A*, the breath rate at the outlet is *U* (with exhalation as positive):

(9) $$U(t)=\frac{dQ}{dT}\cdot \frac{1}{A}=-6.25\sin(\frac{2\pi }{3}t)$$

Due to the fact that only exhalation provides heat to TECE, only exhalation will be studied here. Assuming that the velocity of exhaled gas does not decrease when passing over TECE, and the heat transfer mode is external sweeping flat wall convective heat transfer. Under this premise, the critical Reynolds number is Rec = 5 × 10^5^, the TECE temperature *t_w_* = 27 °C, the gas temperature *t_∞_* = 31 °C, the qualitative temperature *t_m_* = (*t_w_* + *t_∞_*)/2. Then, the thermal conductivity λ, kinematic viscosity ν, and Planck number *Pr* can be determined by examining the physical properties of air at this qualitative temperature. As the Reynolds number is always less than the critical Reynolds number during exhalation, and the boundary layer flow is laminar. Thus, the Nusselt coefficient *Nu* and the average convective heat transfer coefficient *h* of the flat wall can be obtained as follows:

(10) $$Nu=0.664\cdot Re^{1/2}\cdot Pr^{1/3}$$

(11) $$h=\frac{\lambda }{l}Nu$$

**Figure 6:**
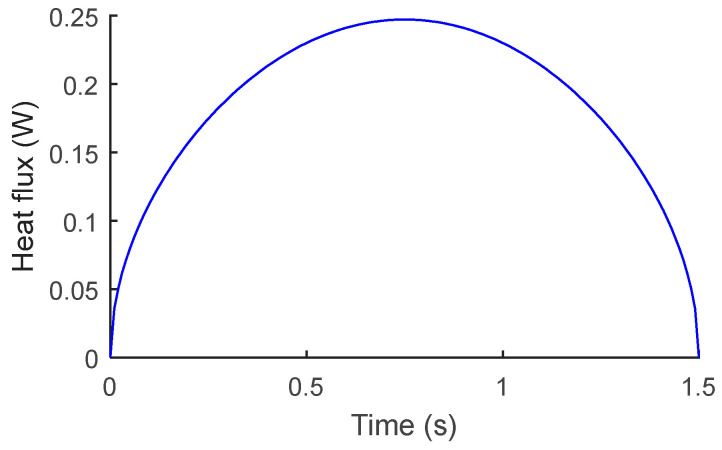
Heat exchange between gas and TECE during breath.

The heat exchange *Φ* between flat wall of TECE and gas during breath, which shown in Figure 6, can be obtained by Newton’s cooling formula:

(12) $$\phi =A\cdot h\cdot (t_{\infty }-t_{w})$$

Total heat transfer *Φ_z_* at expiration can be obtained by integrating heat transfer *Φ* over time t.

(13) $$\phi_{z}=\int \phi (t)dt$$

Ignoring the heat loss from the cold end of the thermoelectric generating unit, the power of the TECE is equal to the input heat *Q_h_* = *Φ_z_*, the input heat will be converted to Fourier heat *Q_f_*, Parthier heat *Q_p_*, and Joule heat *Q_j_*.

(14) $$W=Q_{h}$$

(15) $$Q_{h}=Q_{f}+Q_{p}-Q_{j}$$

(16) $$Q_{f}=K(T_{h}-T_{c})N$$

(17) $$Q_{p}=ST_{h}I=S^{2}T_{h}\frac{(T_{h}-T_{c})N}{R_{i}+R_{L}}$$

(18) $$Q_{j}=I^{2}R_{i}=\frac{S^{2}{\left(T_{h}-T_{c}\right)}^{2}N^{2}}{{\left(R_{i}+R_{L}\right)}^{2}}R_{i}$$

where *R_i_* is the internal resistance of the TECE, so that the external load *R_L_* = 0; The output voltage *V* of the TECE during expiration can be calculated.

**Figure 7:**
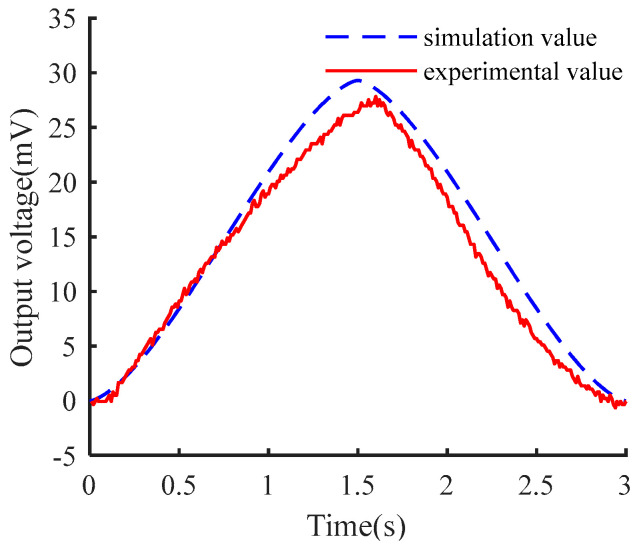
Comparison of experimental and simulated values of TECE.

As shown in Figure 7, with an external temperature of 17.5 °C, a breath gas temperature of 32 °C, and a respiratory cycle of 3.0 s, the peak output voltage is 27.60 mV, while the simulated peak is 29.33 mV, representing an error of 6.25%. This error is due to factors such as contact thermal resistance, thermal radiation, and heat loss at the cold end of the generator.

The TECE, leveraging its superior thermoelectric effect, can fulfill multiple roles including thermoelectric power generation and signal sensing. The direction of its output signal follows the temperature gradient between the two sides. Naturally, reversing the temperature gradient can also reverse the output direction. In mouth breathing applications, it is assumed that the breath gas temperature consistently exceeds the external temperature. This state facilitates the generation of a unilateral fluctuation signal, which then serves as the basis for subsequent tests.

## 4. System Integration and Experimental Verification

### 4.1. System Integration

**Figure 8:**
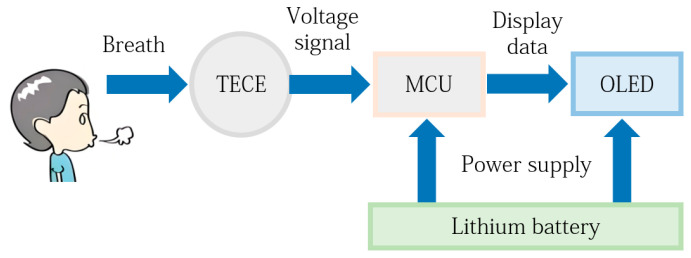
System circuit diagram.

In order to verify the measurement performance of human respiratory rate using the TECE based smart mask, the system architecture of proposed mask shown in Figure 8 is designed and integrated in this paper. The mask mainly consists of Arduino nano core board, OLED display screen, lithium battery, micro TECE, etc. In the overall system of the proposed mask, the TECE is positioned at the mask’s respirator valve, while the screen is placed outside the mask to indicate the rate of respirations. The OLED screen and the TECE are both connected to the Arduino nano core board, which is responsible for signal processing and data transmission to the screen. Additionally, a lithium battery powers the entire system. During each respiration, a temperature difference is generated across the TECE, producing a voltage output to the Arduino core board, which processes the signal and transmits the data to the OLED screen.

**Figure 9:**
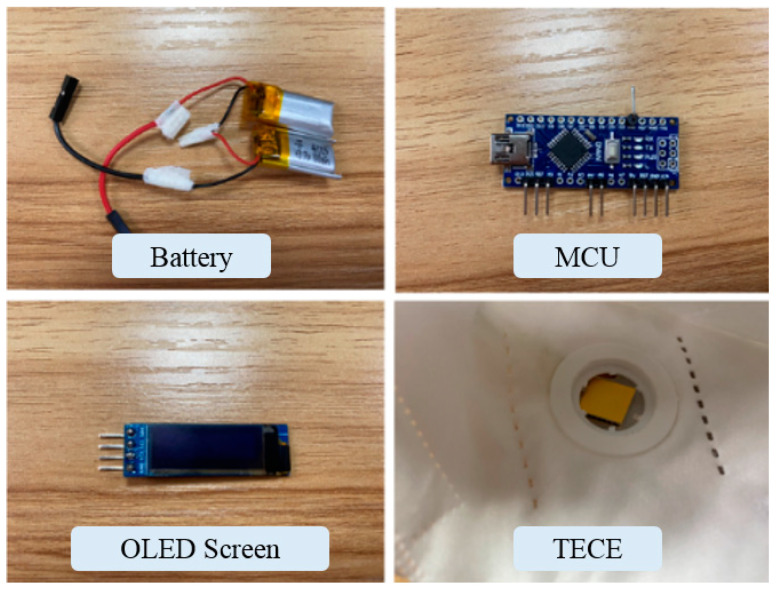
Components of the smart mask.

The required components for the system are illustrated in Figure 9. The Arduino nano core board, featuring the ATMEGA328 chip manufactured by Atmel Corporation (San Jose, CA, USA), is an ultra-compact open-source simple I/O platform, weighing only 6 g. It operates at approximately 5 V DC with a 400 mA current capacity and a 16 MHz clock speed, providing signal processing capabilities; The employed 0.91-inch OLED screen from Geekcreit operates at a supply voltage range of 3.3 V to 5 V, has a four-pin interface, a resolution of 128 × 32 pixels, with IIC communication, and an SSD1306 driver chip manufactured by Solomon Systech Limited (Hong Kong, China). The entire system is powered by a 3.7 V lithium battery. As the supply voltage of the Arduino nano core board is above 5 V, two 3.7 V lithium batteries are connected in series to supply power to the system. The micro TECE with a size of 15 mm × 15 mm serves as the sensing source for the system.

**Figure 10:**
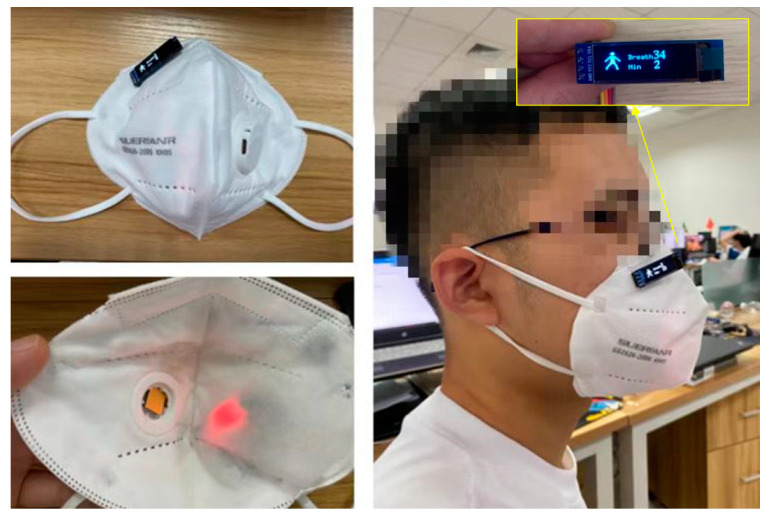
The integrated mask and wearing effect.

After completing the integration of the smart mask, a human wearing effect test was conducted as shown in Figure 10. It can be seen that the mask has a friendly interactive interface, which can automatically count and display the rate of breaths on the OLED screen. Based on this mask, the rate of breaths taken by the human body over a certain period of time can be visually displayed, and the breathing condition of the mask wearer can be determined without the need for other auxiliary tools.

### 4.2. Respiratory Rate Testing

This section mainly focuses on the quantitative analysis of the sensing characteristics of the smart mask, which tested at different breathing rate. During the testing process, one side of the micro TECE was close to the human mouth, while the other side was exposed to the air environment. The output terminal of the TECE was connected to a data acquisition module in ATMEGA328 on the Arduino core board. After wearing the mask, a temperature difference is formed through natural breathing by tester, and the corresponding voltage difference is generated between the two sides of the micro TECE. Subsequently, the amplitude and the frequency of the open circuit voltage of the micro TECE are measured in real time by the data acquisition module.

The experimental conditions are conducted at ambient temperature of 17.5 °C, with exhaled gas temperature around 32 °C. To obtain effective voltage amplitude data, the obtained signals are subjected to mean removal processing. As shown in Figure 10 of the processed voltage waveform, when the respiratory cycle is at 1.9 s, 1.4 s, 0.69 s, and 0.47 s, the output amplitudes of voltage signals by TECE are 18.44 mV, 13.46 mV, 9.42 mV, and 6.07 mV, respectively.

**Figure 11:**
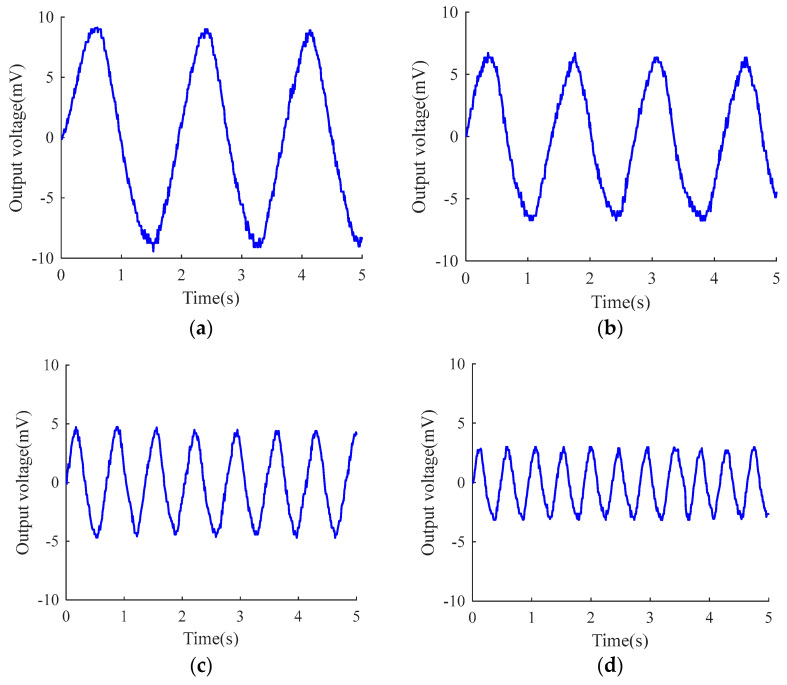
Response curves under different breathing conditions. (**a**) Breathing cycle is 1.9 s. (**b**) Breathing cycle is 1.4 s. (**c**) Breathing cycle is 0.69 s. (**d**) Breathing cycle is 0.47 s.

In Figure 11, it is obvious that as the breathing rate increases, the output voltage amplitude decreases. The main reason for this is that, on the one hand, shortening the breathing cycle leads to lighter breathing, and the intensity of the breath gas heating the TECE is insufficient. On the other hand, temperature transmission requires time, and the faster the breathing rate, the shorter the duration of the breath gas heating the TECE is, resulting in a greater difference between the temperature on the inner side of the TECE and the actual mouth temperature of the human body.

According to above experiment, the proposed smart mask can recognize voltage signals that meet threshold conditions of breaths within a set time, and accurately measurement the respiratory rate of the human body under different breaths conditions by counting the number of recognized breaths. In addition, the mask can use IIC communication to drive the OLED and visually display respiratory rate data without affecting its protective function.

## 5. Conclusions

In this paper, a novel sensible smart mask using micro TECE is proposed, which can detect and display the respiratory rate in real time. The effectiveness of the proposed mask was verified by a human wearing test. Based on the analysis and experiment results, the following conclusions are drawn:

(1) The temperature conversion characteristic of TECE is analyzed, and the analysis exhibited good agreement with experimental results. The results indicated that the output voltage is linearly related to the temperature difference.
(2) The fundamental characteristics of breath are analyzed, the heat conduction relationship during human respiration is studied, and the output law of TECE due to human respiration is determined through simulation and experiment.
(3) A sensible smart mask, which can show respiratory rate in real time, is developed by integrating MCU and OLED module, and the effectiveness and accuracy of the proposed sensible smart mask are verified by human respiratory rate experiments.

Overall, this paper introduces a new smart mask utilizing the thermoelectric effect to detect respiratory rate. This mask can visually display breathing information, which is particularly useful during special periods. Additionally, the proposed mask offers advantages in compact size, lightweight, and facial conformity. However, to ensure wearing comfort, this study employed a smaller TECE, thus a self-powered respiratory rate collection system was not realized. In subsequent stages, the conductive material of the TECE can be optimized by modifying its thickness to enhance its output power and efficiency. Further exploration into the impact of environmental factors like temperature and humidity on TECE performance is also warranted to improve its stability.

## Data Availability

The data that support the findings of this study are available from the corresponding authors upon reasonable request.
